# Therapeutic Approaches with Iron Oxide Nanoparticles to Induce Ferroptosis and Overcome Radioresistance in Cancers

**DOI:** 10.3390/ph18030325

**Published:** 2025-02-26

**Authors:** Dorianne Sant’Angelo, Géraldine Descamps, Valentin Lecomte, Dimitri Stanicki, Sébastien Penninckx, Tatiana Dragan, Dirk Van Gestel, Sophie Laurent, Fabrice Journe

**Affiliations:** 1 Department of Human Biology and Toxicology (Cancer Research Unit), Faculty of Medicine, Research Institute for Health Sciences and Technology, University of Mons (UMONS), 7000 Mons, Belgium; 2 Laboratory of Clinical and Experimental Oncology (LOCE), Institute Jules Bordet, HUB, Université Libre de Bruxelles (ULB), 1070 Brussels, Belgium; 3 Department of General, Organic and Biomedical Chemistry, NMR and Molecular Imaging Laboratory, University of Mons (UMONS), 7000 Mons, Belgium; geraldine.descamps@umons.ac.be (G.D.); valentin.lecomte@umons.ac.be (V.L.); dimitri.stanicki@umons.ac.be (D.S.); sophie.laurent@umons.ac.be (S.L.); 4 Department of Medical Physics, Institut Jules Bordet, HUB, Université Libre de Bruxelles (ULB), 1070 Brussels, Belgium; sebastien.penninckx@hubruxelles.be; 5 Department of Radiotherapy, Institute Jules Bordet, Hopital Universitaire de Bruxelles (HUB), Université Libre de Bruxelles (ULB), 1070 Brussels, Belgium; tatiana.dragan@hubruxelles.be (T.D.); vangesteldirk@netscape.net (D.V.G.)

**Keywords:** metallic nanoparticles, ferroptosis, head and neck cancers

## Abstract

The emergence of nanotechnology in medicine, particularly using iron oxide nanoparticles (IONPs), may impact cancer treatment strategies. IONPs exhibit unique properties, such as superparamagnetism, biocompatibility, and ease of surface modification, making them ideal candidates for imaging, and therapeutic interventions. Their application in targeted drug delivery, especially with traditional chemotherapeutic agents like cisplatin, has shown potential in overcoming limitations such as low bioavailability and systemic toxicity of chemotherapies. Moreover, IONPs, by releasing iron ions, can induce ferroptosis, a form of iron-dependent cell death, which offers a promising pathway to reverse radio- and chemoresistance in cancer therapy. In particular, IONPs demonstrate significant potential as radiosensitisers, enhancing the effects of radiotherapy by promoting reactive oxygen species (ROS) generation, lipid peroxidation, and modulating the tumour microenvironment to stimulate antitumour immune responses. This review explores the multifunctional roles of IONPs in radiosensitisation through ferroptosis induction, highlighting their promise in advancing treatment for head and neck cancers. Additional research is crucial to fully addressing their potential in clinical settings, offering a novel approach to personalised cancer treatment.

## 1. Clinical Context and Objectives

Cancer remains one of the most challenging diseases, accounting for a significant number of deaths worldwide. According from GLOBOCAN database, in 2022, nearly 20 million new cases were reported and 9.7 million deaths were recorded [1]. Europe is particularly affected by this burden as it represents less than 10% of the population but accounts for 20.4% of cancer-related deaths. It is estimated that around 50% of patients suffering from cancer will need radiotherapy. The number of patients needing this treatment is expected to increase with the increasing incidence of new cases of cancer in the future [2]. Head and neck cancers (HNCs) are the seventh most common cancer and also benefit from radiotherapy. In 2022, more than 940.000 cases of HNC were reported and more than 480.000 deaths were recorded [1]. Despite advances in understanding HNC biology (EGFR overexpression and p53 mutation), and the evolution of therapeutic strategies (chemoradiation treatment, targeted therapy, and immunotherapy) [3,4,5], these cancers often exhibit radioresistance, leading to treatment failure and poor prognoses.

Radiotherapy is essential in HNC treatment, as the sole treatment and alternative to surgery in early stages (I and II), and as part of a combined approach with cisplatin, radically or adjuvant to surgery in locally advanced stages (III and non-metastatic IV). Combination therapy in the latter stages is effective in over 50% of patients. However, HNCs are challenging due to the anatomical complexity of the region, involving critical structures at the intersection of the digestive and respiratory systems and the vocal cords. Despite significant advancements in radiotherapy in recent years, side effects remain common and often debilitating. A prominent example is xerostomia, a persistent dry-mouth condition that severely impacts patients’ quality of life [6]. Another major limitation is the difficulty in re-irradiating the same area. Indeed, re-exposure to radiation significantly increases toxicity risks, even though it can improve overall survival [7]. Furthermore, these cancers are frequently associated with the development of new primary tumours in the same region. These tumours arise due to continued exposure to known risk factors, such as the combined consumption of tobacco and alcohol [8,9,10], making re-irradiation even more challenging.

In this context, the development of radiosensitising agents is of critical importance. These agents have the potential to enhance the efficacy of treatments while possibly reducing the required radiation dose, thereby mitigating toxic side effects. Innovative strategies using nanoparticles (NPs) targeting tumours in combination with radiotherapy have been developed and have already shown promise in sensitising cancer cells to radiation, improving tumour control. The effect has been demonstrated using multiple nano-objects, including those based on gold, platinum, gadolinium, and hafnium in a wide variety of tissues [11]. These preclinical results have paved the way for an initial positive phase II study. The Act.In.Sarc trial (NCT02379845) demonstrated that the nanoparticle NBTXR3 (hafnium oxide), when activated by preoperative radiation therapy, doubled the rate of pathologic complete response after resection compared with preoperative radiotherapy alone in adult patients with locally advanced soft tissue sarcoma of the extremity or trunk wall (16.1% vs. 7.9%). Moreover, NBTXR3 did not impact the patients’ quality of life, notably in terms of late-onset adverse events such as fibrosis, oedema, and joint stiffness [12].

Our ongoing research has focussed on iron oxide nanoparticles (IONPs), which have demonstrated both radiosensitising properties [13] and use as magnetic resonance (MRI) contrast agents [14]. Although clinical trials of HNCs involving IONPs are still limited, a study currently underway is evaluating the effect of hafnium oxide nanoparticles combined with radiotherapy and immunotherapy (NCT04862455), highlighting the relevance of using metallic nanoparticles in combination with current treatments for HNCs.

Additionally, the concept of ferroptosis, a unique form of regulated cell death triggered by toxic lipid peroxidation, has recently gained attention in cancer therapy [15]. Ferroptosis is linked with iron metabolism and the accumulation of reactive oxygen species (ROS), ultimately leading to cell death.

In this review, we specifically focus on the properties of iron oxide nanoparticles and their therapeutic effects on HNC cells. We also explore their role in the induction of ferroptosis, which represents a vulnerability in HNCs. Finally, we examine the known effects of combining iron oxide nanoparticles with radiotherapy and propose this therapeutic regimen in the context of HNCs, as there are already few studies on the subject, all suggesting great promise for improving treatment outcomes.

## 2. Ferroptosis

### 2.1. History

Damage induced by lipid peroxidation has been recognised for decades in relation to neuronal damage [16], increased cancer risk [17,18], and ischemia/reperfusion injury [19] and has been extensively described since then. The term ferroptosis appeared much later, and the concept of toxic lipid peroxidation has been implicated in other human pathologies including cancers.

In 2003, Dolma et al. conducted a high-throughput screening aimed at uncovering new compounds capable of inducing cell death in genetically modified tumour cells. This screening yielded several promising molecules, including one that demonstrated the ability to induce cell death specifically in a mutant cell line but not in the wild-type cell line. This cell line harboured a mutation of the *HRAS* (*RAS^V12^*) oncogenic gene and also expressed the small T oncoprotein from simian virus 40. This molecule was named erastin, in reference to “eradicator of RAS- and small T oncoprotein (ST)-expressing cells”, and was revealed to trigger cell death through a non-apoptotic pathway [20]. Building upon this discovery, in 2008, the team conducted another screening which led to the identification of an RAS-selective lethal (RSL) compound, also capable of inducing cell death in oncogenic RAS-mutant cell lines via a non-apoptotic mechanism [21].

By 2012, the same group developed a deeper understanding of this cell death pathway activated by erastin and RSL3, suggesting the term ferroptosis to characterise this novel form of cell death [22]. Further insights regarding the initial observations on ferroptosis pathways are described in the work by Tal Hirschhorn and Brent R Stockwell [23].

### 2.2. Morphological Features

The researchers at Stockwell’s laboratory were pioneers in studying the morphological features of cells undergoing ferroptosis. This pathway presents a distinctive phenotype compared to other forms of cell death. Unlike apoptosis, there is no release of cytochrome c from mitochondria, no activation of caspases, and no staining with annexin V. Additionally, the nuclei maintain their structure without undergoing morphological changes such as chromatin condensation or fragmentation [20,22,24,25]. Ferroptotic cells also differ from necrotic cells by the absence of cytoplasmic swelling and direct plasma membrane rupture [22]. Furthermore, ferroptosis can be distinguished from autophagy by the lack of double-membrane vesicles [22] and from pyroptosis by the absence of blebbing and early plasma membrane rupture [25,26]. Notably, ferroptotic cells display smaller mitochondria with increased membrane density [22]. At the molecular level, ferroptosis diverges from other regulated cell death pathways, as it is triggered by specific inducers and does not involve the same cellular machinery [27].

### 2.3. Ferroptotic Markers

Ferroptotic markers are biological indicators or characteristics associated with the occurrence or progression of ferroptosis. These markers are molecular, cellular, or histological features which indicate the presence or extent of ferroptosis in various biological systems, including cancer. Common ferroptotic markers are lipid peroxidation, glutathione depletion, iron accumulation, mitochondrial dysfunction, oxidative stress, and altered expression of ferroptosis-related genes, such as *SLC7A11* (solute carrier family 7 member 11), *GPX4* (glutathione peroxidase 4), and *FTH1* (ferritin heavy polypeptide 1) [22,25,28].

### 2.4. Ferroptosis Mechanisms

Ferroptosis refers to iron-dependent cell death. It is driven by the intracellular accumulation of iron, which promotes the production of reactive oxygen species (ROS) through the Fenton reaction, leading to excessive lipid peroxidation of the cell membranes. These processes ultimately cause cell death as a result of toxic lipid damage.

#### 2.4.1. Iron Metabolism

**Figure 1:**
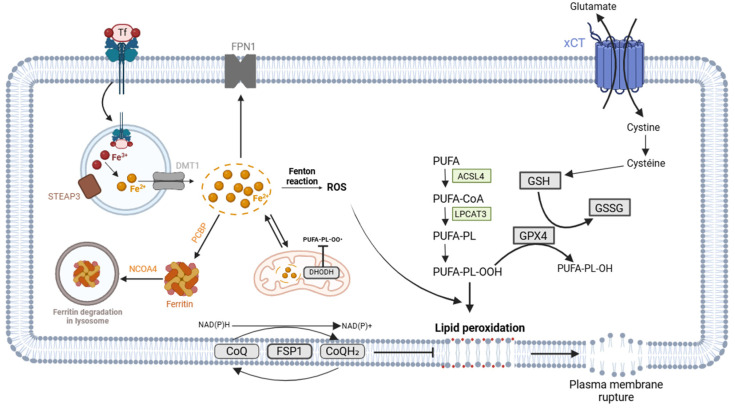
Overview of the different pathways involved in the induction of ferroptosis. Ferroptosis is a cell death pathway dependent on iron metabolism, lipid metabolism, and ROS metabolism. ROS production through the Fenton reaction can lead to the conversion of phospholipids hydroperoxide into phospholipid hydroperoxy radicals and promote excessive lipid peroxidation. Red dots: Fe^3^⁺ ions, yellow dots: Fe^2^⁺ ions, Tf: transferrin, STEAP3: STEAP3 metalloreductase, DMT1: divalent metal transporter 1, labile iron pool: represented in yellow and contains Fe^2^⁺, PCBP: poly (rC)-binding protein 1, NCOA4: nuclear receptor coactivator 4, ROS: reactive oxygen species, DHODH: dihydroorotate dehydrogenase (quinone), FSP1: ferroptosis suppressor protein 1, CoQ: coenzyme Q (ubiquinone), CoQH2: reduced form of coenzyme Q10 (ubiquinol), PUFAs: polyunsaturated fatty acids, PUFA-PLs: PUFA phospholipids, PUFA-PL-OOH: phospholipid hydroperoxide, PUFA-PL-OH: phospholipid alcohol, ACSL4: acyl-CoA synthetase long-chain family member 4, LPCAT3: lysophosphatidylcholine acyltransferase 3, xCT: cystine/glutamate antiporter, GSH: reduced glutathione, GSSG: oxidised glutathione, GPX4: glutathione peroxidase 4, BH4: tetrahydrobiopterin. Created using BioRender (https://www.biorender.com/).

In physiological conditions, iron primarily enters cells bound to transferrin (Figure 1). This iron–transferrin complex binds to transferrin receptor 1 on the cell surface and is internalised by endocytosis [29]. Within the endosome, Fe^3+^ is released and then reduced to Fe^2+^ by endosomal ferrireductase [29]. Fe^2+^ is subsequently transported into the cytoplasm through the divalent metal transporter 1 (DMT1) and is included in the labile iron pool [29]. It can be (1) utilised for cellular processes and incorporated into iron-containing proteins, (2) exported by ferroportin 1 (FPN1) and then reoxidised to Fe^3+^ by ferroxidase [30], or (3) stored in ferritin if in excess [29].

Importantly, storage in ferritin is crucial to preventing redox reactions with iron. To this end, Fe^2+^ enters ferritin and is oxidised into Fe^3+^, which is then stored in the cavity of ferritin [29]. Chaperone proteins known as poly (rC)-binding proteins (PCBPs 1–4) mediate the transport of iron into ferritin [29]. Iron release occurs through the degradation of ferritin in the lysosome, a mechanism termed ferritinophagy, which is part of the autophagy process [31]. Nuclear receptor coactivator 4 (NCOA4) serves as a receptor and binds to ferritin for its delivery to the lysosome [31].

Iron can contribute to the accumulation of ROS through various mechanisms. Firstly, iron and its derivatives can be incorporated into proteins involved in ROS production, such as NADPH oxidase, lipooxygenases, cytochrome P450 enzymes, and enzymes from the electron transport chain in the mitochondria [32]. Iron pools are present in the lysosomes, the mitochondrial matrix, and the cytosol, where they can initiate the Fenton reaction [32], which catalyses the formation of Fe^3+^ from Fe^2+^ and hydrogen peroxide (H_2_O_2_) and generates hydroxide (OH-) and hydroxyl radicals (OH•) [33,34].

It is worth noting that iron overload is associated with metabolic diseases such as haemochromatosis, a condition in which excessive iron is absorbed through the intestine and cannot be effectively eliminated by the body. This leads to organ damage, most commonly affecting the liver, but it can also lead to diabetes and impact the gonads and heart [35]. Additionally, iron uptake itself can be linked to various side effects, including gastrointestinal disorders such as nausea, vomiting, diarrhoea, constipation, and abdominal pain. Other side effects may include skin rashes and blood pressure abnormalities [36,37]. These side effects could potentially be prevented or reduced through the use of targeted IONPs, which will be discussed later in this review.

#### 2.4.2. Lipid Peroxidation Process

Lipid oxidation by ROS, also termed peroxidation, occurs in three distinct steps wherein peroxide products are generated, maintain the reaction, and induce damage [38]. The first target of ROS is polyunsaturated fatty acids (PUFAs), mainly due to the presence of unstable carbon–carbon double bonds between methylene groups [34]. This process is pivotal in ferroptosis, particularly targeting arachidonic acid and adrenic acid, the main PUFAs susceptible to peroxidation [30,39]. An excessive rate of lipid peroxidation can deplete cellular antioxidant capacities, ultimately triggering programmed cell death [34].

The three steps of lipid peroxidation are initiation, propagation, and termination. Initiation begins with the removal of a hydrogen atom from a PUFA by free radicals such as OH•, resulting in the formation of a lipid radical. In the propagation step, lipid radicals form lipid peroxy radicals, which further react with another adjacent lipid to produce a lipid radical and lipid hydroperoxide. Finally, antioxidant molecules become involved in the termination phase by reacting with lipid peroxy radicals and stopping the process [34,38]. Peroxidation products, such as lipid hydroperoxide, malondialdehyde (MDA), and 4-hydroxy-2-nonenal (4-HNE), can induce cell death by reacting with biomolecules [34]. Moreover, lipid peroxidation can lead to changes in cell membrane integrity and functionality. It can alter membrane fluidity, permeability, lipid–lipid and lipid–protein interactions, and ion gradients. Additionally, it can impact the initiation of signalling pathways [40,41]. Of note, as mentioned by Tang et al. and Liu et al., the precise end-effectors of the ferroptotic pathway remain unidentified. Since ferroptotic cell death leads to cell lysis, the identification of proteins involved in pore-forming membrane activities needs to be elucidated, along with an exploration of the roles played by the aforementioned peroxidation products [30,42].

Very recently, Scott J. Dixon and James A. Olzmann summarised the mechanisms leading to cell membrane rupture. The increase in lipid peroxidation causes an increase in membrane tension that activates ion channels sensitive to these changes. These events will induce unregulated ion efflux and loss of ionic homeostasis and eventually rupture the cell membrane after nanopore formation and cell swelling [43].

Ferroptosis is not regulated by the same factors as other cell death pathways, but it appears to be promoted by autophagy. This is evident from the turnover of ferritin, the primary iron storage protein, within the autophagy process. Additionally, autophagic effectors have been observed to influence ferroptosis induction [30,42,44]. However, as mentioned in a recent review by Chen et al., several pathways within autophagy can either enhance ferroptosis or have cytoprotective effects against it [45].

Mitochondria are protected from membrane lipid peroxidation through the storage of iron by ferritin and antioxidant systems (notably DHODH, as explained in Section 2.5.4). Through their essential role in maintaining ROS balance, mitochondria are also involved in the induction of ferroptosis. A detailed exploration of the role each organelle plays in ferroptosis can be found in the reviews [43,46], and specific information regarding mitochondria can be found in the articles [47,48,49].

### 2.5. Antioxidant Defence Systems Against Ferroptosis

Ferroptosis is a complex, iron-dependent cell death pathway driven by ROS production and lipid peroxidation, with key mechanisms involving disrupted iron and lipid metabolism. Understanding these pathways and their regulation could open potential therapeutic strategies. Multiple antioxidant systems have been shown to play a role in preventing ferroptosis. Understanding these protective pathways offers valuable insights into potential therapeutic strategies for diseases involving ferroptotic cell death, particularly by targeting these key antioxidant mechanisms.

#### 2.5.1. Xc^−^/GSH/GPX4 Pathway

The selenoprotein glutathione peroxidase 4 (GPX4) is the main enzyme involved in antioxidant defence mechanisms [50] and protects cells against lipid peroxidation [51]. GPX4 functions by reducing lipid hydroperoxides (LOOHs) into corresponding nontoxic hydroxy derivatives (LOH) through the oxidation of GSH to GSSG [39,50] (Figure 1). Specifically, the selenol group in GSH is oxidised into selenic acid along with the reduction in toxic lipids into nontoxic alcohol. Subsequently, selenic acid is reduced back to selenol with two molecules of GSH [52]. Notably, GPX4 is the only GSH peroxidase capable of reducing phospholipid hydroperoxides present in the membrane [52].

Thus, the Xc^−^/GSH/GPx4 pathway regulates ferroptosis. Cystine is transported into the cell by the antiporter Xc^−^ in exchange for intracellular glutamate [52]. Cystine is then reduced to cysteine, a precursor of GSH [52], and this reaction is mainly catalysed by thioredoxin reductase 1 (TXNRD1) [53]. GPX4 then utilises GSH as a cofactor for its antioxidant activity [52]. Inhibition of the Xc^−^ antiporter, its subunit SLC7A11, GSH synthesis, or GPX4 activity all promote ferroptosis. Moreover, GPX4 protects cells from ROS formation induced by Fe^2+^ by converting lipid hydroperoxides into nontoxic lipid alcohols [54].

#### 2.5.2. NAD(P)H/FSP1/CoQ10 Pathway

Olzmann and his team identified ferroptosis suppressor protein 1 (FSP1), previously known as apoptosis-inducing factor mitochondrial 2 (AIFM2), as an additional pathway which prevents lipid peroxidation and protects cells from ferroptosis [55]. Indeed, FSP1 and GSH/GPX4 axes are parallel pathways that operate to reduce toxic lipid peroxidation.

FSP1 is recruited to lipid droplets and to the plasma membrane [55], serving as a biomarker of ferroptosis resistance [55]. FSP1 acts as an oxidoreductase and reduces CoQ10 (ubiquinone) to CoQ10H2 (ubiquinol) using NAD(P)H/H^+^ as an electron source [55,56] (Figure 1). CoQ10H2 operates as an antioxidant to prevent lipid peroxidation and can indirectly generate α-tocopherol (vitamin E) [56], which further reduces lipid radicals [55]. CoQ10 is generated from isopentenyl pyrophosphate via the mevalonate pathway. Interestingly, inhibiting the rate-limiting enzyme (HMG-CoA reductase) of the mevalonate pathway has been shown to sensitise cells to ferroptosis [53]. NADPH plays a crucial role in ferroptosis as an electron donor to the GSH and thioredoxin systems, as well as for mevalonate synthesis [53].

There seems to be another pathway that protects cells from ferroptosis and is dependent on FSP1. This protein can promote the endosomal sorting complex required for transport III (ESCRT-III), which is notably involved in membrane repair [56] and thereby prevents ferroptosis.

#### 2.5.3. BH4

Tetrahydrobiopterin (BH4) seems to be part of another GPX4-independent pathway that can inhibit ferroptosis (Figure 1). BH4 is a cofactor for various enzymes, is involved in the synthesis of some neurotransmitters, and is a free-radical-trapping molecule [57,58]. BH4 thereby prevents autooxidation in lipid membranes [59]. The enzyme GTP cyclohydrolase-1 (GCH1) is the rate-limiting enzyme in the synthesis of BH4. BH4 can be recycled by dihydrofolate reductase (DHFR) [57,58]. DHFR also supports the anti-ferroptotic activity of BH4 [59].

#### 2.5.4. DHODH and CoQ10/CoQH2

Another pathway which prevents lipid peroxidation accumulation and ferroptosis is found in the mitochondria. Indeed, dihydroorotate dehydrogenase (DHODH) acts in parallel with mitochondrial GPX4 in order to reduce mitochondrial lipid peroxidation (Figure 1). DHODH acts by reducing CoQ into CoQH2 [60], which is an antioxidant molecule that inhibits initiation and propagation in lipid peroxidation [61]. This new ferroptosis defence mechanism was first reported by Chao Mao et al. [60].

#### 2.5.5. Other Antioxidant Pathways

Other antioxidant systems can prevent ferroptosis. These include enzymes responsible for the detoxification of secondary products, like 4-HNE, or through reshaping the composition of the plasma membrane, as explained in the review of Punziano et al. [62].

Ferroptosis is a specific form of cell death involving precise induction mechanisms, including increased ferrous iron, ROS imbalance, and lipid peroxidation. Understanding the primary pathways leading to ferroptosis is essential for developing therapeutic strategies and interventions. The Section 2.7 will discuss the molecular targets of ferroptosis that have already been studied in HNCs.

### 2.6. Ferroptotic Pathways and Involvement in Head and Neck Cancers

Several of the molecular pathways mentioned above are particularly interesting as potential targets in the context of HNCs, as they serve as markers for survival prognosis. The key targets are summarised in Table 1, identified through data from The Human Protein Atlas, specifically using The Cancer Genome Atlas (TCGA) dataset and RNA expression profiles [63]. Targeting the associated overexpressed proteins presents a promising approach to inducing cell death specifically in this type of cancer. Indeed, other studies have identified these proteins as prognostic markers: high thioredoxin has been shown to correlate with poorer survival in tongue carcinoma (*p* < 0.05) [64]; positive staining (determined by a specific established score in the review) of SLC7A11 is associated with poorer survival in laryngeal carcinoma (*p* < 0.05) [65]; and high expression of FTH1 is linked to poorer overall survival (*p* < 0.01) and worse disease-free survival (*p* < 0.001) in HNCs [66].

### 2.7. Pharmacological Inducers and Inhibitors of Ferroptosis in Head and Neck Cancers

Several molecules can be used to induce ferroptosis. It is generally described that ferroptosis inducers can be classified according to their targets: system Xc^−^ (xCT or specifically subunit SLC7A11), GSH synthesis, GPX4, and others such as NRF2. There are multiple research projects focussing on ferroptosis induction in HNCs. This review presents the pharmacological molecules that have been shown to play a role in inducing/inhibiting ferroptosis in such cancers. Indeed, ferroptosis emerges as a promising avenue in HNC therapy, underscored by the intricate interplay between NRF2 inhibition and downstream pathways, such as FTH1-mediated iron sequestration, further accentuating the significance of targeting iron accumulation and ferritinophagy for ferroptosis induction. Additionally, the differential response of HNC cells to ferroptosis inducers and the multifaceted roles of molecules like FSP1 and SLC7A11 emphasise the complex landscape of ferroptosis regulation, offering new possibilities for combinatory strategies in HNC treatment.

#### 2.7.1. NRF2 Inhibition and Its Downstream Pathway

**Figure 2:**
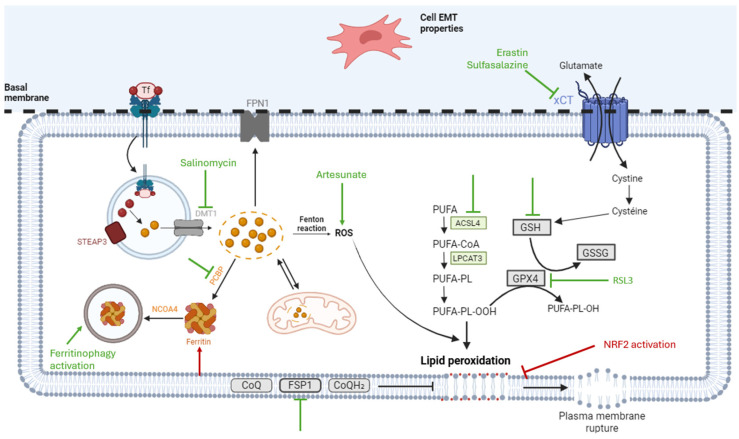
Schematic representation of the molecular mechanisms of ferroptosis inducers and repressors in head and neck cancer cell models. The inducers are represented in green and include artesunate (indirectly increases ROS production), salinomycin (silences transporter DMT1) and erastin or sulfasalazine (inhibit transporter xCT). Knockdown of PCBP1 stimulates ferritinophagy. Cells undergoing epithelial–mesenchymal transition (EMT) seem to be more sensitive to ferroptosis inducers. The inhibitors are represented in red and include the activation of NRF2 and stimulation of iron storage through ferritin. RSL3: RAS-selective lethal small molecule 3. Created using BioRender (https://www.biorender.com/).

A treatment strategy largely explored in the literature for various cancers is the inhibition of NRF2. Roh et al. investigated the ability of artesunate (an antimalarial drug) to induce ferroptosis in cisplatin-resistant HNCs by promoting ROS accumulation and depleting GSH (Figure 2). However, some resistant HNC cells were less sensitive to artesunate due to the activation of the NRF2/ARE pathway, which confers protection against ferroptosis. Combining artesunate with NRF2/ARE pathway inhibition proved to be an effective approach for inducing cell death in such resistant HNC cells [67]. More recently, Shin et al. demonstrated that resistance to the GPX4 inhibitor RSL3 (RAS-selective lethal 3) could be attributed to NRF2 activation. Treatment with RSL3 induced endoplasmic reticulum (ER) stress, resulting in increased p62 expression. After ER stress, the p62–Keap1 interaction activates NRF2, inducing the transcription of genes containing antioxidant response elements (AREs) [68]. Downstream targets of AREs include *FTH1*, *FPN*, and *HO-1*, which act to reduce free iron in the cytoplasm or enhance antioxidant defences, thereby conferring protection against ferroptosis [68] (Figure 2).

#### 2.7.2. Strategies Targeting Iron Accumulation and Ferritinophagy

Liu et al. observed variations in the sensitivity of HNCs to ferroptosis inducers. High levels of *FTH1*, the heavy chain of ferritin, which is also a target gene of NRF2, were found to reduce sensitivity to ferroptosis inducers, such as erastin and RSL3, by facilitating iron storage and consequently preventing ferroptosis [69] (Figure 2).

Lee and Roh demonstrated that the silencing of divalent metal transporter 1 (DMT1) or the use of salinomycin (a selective anti-cancer stem cell agent, traditionally used as an anti-coccidial drug) could induce ferroptosis by promoting iron accumulation (Figure 2). DMT1, located on the lysosome membrane, facilitates the transport of Fe^2+^. The inhibition of DMT1, either through silencing or salinomycin treatment, results in iron sequestration within the lysosome and triggers an iron starvation response in the cytoplasm. Consequently, the cellular response leads to an increase in the labile iron pool, the activation of the Fenton reaction, and the production of hydroxyl radicals, all of which are prerequisites for lipid peroxidation and ferroptosis [70].

Cystine deprivation through xCT inhibition by sulfasalazine leads to reduced GSH, increased lipid peroxidation, and increased ferroptosis (Figure 2). This effect was shown to be dependent on the activity of the glutaminolysis pathway. The dihydrolipoamide dehydrogenase (DLD) enzyme is part of the KGDH complex (itself part of the glutaminolysis pathway), which can produce ROS molecules and impact lipid peroxidation. Therefore, both DLD inhibition and glutaminolysis inhibition prevent the effects of cystine deprivation on the induction of ferroptosis. A lack of cystine increases intracellular Fe^2+^ accumulation via two pathways: (1) stimulation of KGDH activity that eventually leads to mitochondrial lipid peroxidation and iron accumulation and (2) upregulation of the iron starvation response [71]. It has to be highlighted that the glutaminolysis pathway seems to be connected with the induction of ferroptosis.

Cisplatin-resistant HNC cells are less sensitive to ferroptosis inducers. However, the combination of the ferroptosis inducer sulfasalazine and suppression of glutaredoxin 5 (GLRX5) shows an increase in intracellular free iron, lipid peroxidation, and then ferroptosis. GLRX5 is a mitochondrial protein that plays an important role in iron homeostasis [72].

Finally, targeting poly (rC)-binding protein 1 (PCBP1) represents another potential strategy to induce ferroptosis in HNCs. PCBP1 regulates ALOX15 and BECN1, both of which play crucial roles in PUFA generation and ferritinophagy, respectively. Therefore, the knockdown of PCBP1 promotes ferritinophagy, enhances Fe^2+^ accumulation, induces mitochondrial dysfunction and ROS production, and facilitates oxidised PUFA generation [73], all essential steps for triggering ferroptosis (Figure 2).

#### 2.7.3. Mesenchymal Phenotype

Erlotinib-tolerant persister HNC cells exhibit increased mesenchymal traits and a metabolic switch toward glutaminolysis, rendering them more susceptible to ferroptosis inducers. Erlotinib is an inhibitor of the epidermal growth factor receptor (EGFR) tyrosine kinase. This sensitivity may be attributed to the KDM5A/MPC1 axis regulation in these cancer cells. Indeed, mitochondrial pyruvate carrier 1 (MPC1) plays a pivotal role in transporting pyruvate into mitochondria, a critical step in oxidative phosphorylation, and also regulates epithelial–mesenchymal transition (EMT). Erlotinib-tolerant persister cells demonstrated reduced MPC1 expression, and the inhibition of MPC1 subsequently increased glutaminolysis, leading to elevated mitochondrial ROS production and increased susceptibility to ferroptosis inducers [74].

Another study revealed HNC cells with mesenchymal properties to be more responsive to ferroptosis inducers (Figure 2). EMT regulation can be achieved through epigenetic mechanisms. For instance, resveratrol activates the histone deacetylase sirtuin 1, resulting in increased expression of zinc finger E-box-binding homeobox 1 (ZEB1), a transcription factor involved in EMT [75].

In a model of salivary adenoid cystic carcinoma, the monoclonal antibody OMP-52 M51 was able to bind NOTCH1, inhibited its subsequent pathway, suppressed tumour growth, and mitigated EMT. Moreover, this antibody exhibited the ability to induce ferroptosis and sensitise cells to the ferroptosis inducer erastin. This represents an interesting strategy for this type of HNC [76].

#### 2.7.4. FSP1 and ACSL4

Recent studies have highlighted elevated levels of FSP1 in drug-tolerant persister HNC cells (describing a group of cells that survives from primary therapy and are responsible for drug resistance) with resistance to cisplatin, along with activation of the FSP1/ACSL4 axis. Combining an FSP1 inhibitor with cisplatin led to a significant reduction in tumour size in a patient-derived xenograft (PDX) mouse model [77].

More recently, Xu et al. identified SUMO-specific peptidase 1 (SENP1) as a predictive biomarker for HNC treatment. They observed that the overexpression of SENP1 in HNCs was correlated with disease progression. SENP1 influences the stability of ACSL4 through deSUMOylation, thereby promoting GPX4 activity and inhibiting ferroptosis. Silencing SENP1 enhances ACSL4 stability, suppresses GPX4 activity, and promotes lipid peroxide accumulation, consequently inducing ferroptosis [78]. Of note, ACSL4 plays a crucial role in converting fatty acids to fatty acyl-CoA esters [56].

The fat mass- and obesity-related gene (*FTO*) functions as a demethylase, erasing N6-methyladenosine (m^6^A) epigenetic modification in mRNA. FTO has been demonstrated to remove m^6^A modifications from ACSL3 and GPX4 mRNA, known as anti-ferroptotic factors, thereby promoting ferroptosis by decreasing the stability of these mRNAs. FTO represents a potential therapeutic target for oral squamous cell carcinoma [79].

#### 2.7.5. SLC7A11 (xCT)

The combination of cisplatin and RSL3 at low concentrations was found to induce significant cell death, with cisplatin enhancing ferroptosis caused by RSL3 (Figure 2). This effect may be attributed to the increased expression of mutant p53 induced by the chemotherapeutic agent. This phenomenon could be explained by the subsequent suppression of SLC7A11, as directly highlighted by Ye et al. [80].

In this context, You et al. showed that paclitaxel-tolerant persister cancer cells were more sensitive to ferroptosis inducers targeting xCT but less sensitive to ferroptosis inducers such as RSL3. This sensitivity shift was accompanied by an increase in fatty acid oxidation and an upregulation of the PGRMC1 protein. PGRMC1 silencing in paclitaxel-tolerant persister cells decreased the sensitivity to ferroptosis inducers. Additionally, it was observed that PGRMC1 induced lipophagy and autophagy, suggesting that altered lipid metabolism could promote ferroptosis [81].

Furthermore, overexpression of the short non-coding RNA miR-34c-3p was shown to downregulate the expression of SLC7A11 and induce an increase in ferroptotic markers [82].

#### 2.7.6. Other Inducers/Inhibitors

The growth factor epiregulin functions as a ligand of EGFR. Silencing epiregulin in combination with cetuximab (monoclonal antibody against EGFR) has been demonstrated to induce Fe^2+^ accumulation, elevate lipid peroxide levels, and downregulate GPX4 in HNCs [83]. These findings suggest that the loss of epiregulin could serve as a predictive marker for sensitivity to ferroptosis following cetuximab treatment, indicating the potential benefit of combining cetuximab with ferroptosis inducers in such patients.

Other molecules were found to induce ferroptosis in a nasopharyngeal cell line, including cucurbitacin B [84] and the plant-derived triterpenoid lupeol [85]. In addition, silencing adipocyte enhancer-binding protein 1 (AEBP1) predisposed cisplatin-resistant oral cancer cells to ferroptosis inducers in vitro [86], and ascorbic acid can induce ferroptosis in a model of oropharyngeal carcinoma [87].

Altogether, targeting ferroptosis pathways through pharmacological inducers and inhibitors offers promising therapeutic opportunities for HNCs, especially by modulating key regulators like NRF2, SLC7A11, FSP1, and GPX4. Strategies such as iron accumulation, glutaminolysis inhibition, and the suppression of protective pathways (i.e., NRF2 activation and FSP1) support the potential for combination therapies to overcome radiotherapy resistance and improve treatment outcomes in HNCs.

## 3. Radiotherapy

Head and neck cancers (HNCs) are predominantly treated with radiotherapy, often serving as a primary or adjuvant therapy depending on the stage and location of the tumour. Radiotherapy is effective in targeting cancer cells while preserving surrounding healthy tissues, making it a cornerstone in the management of HNCs. It is commonly combined with surgery or chemotherapy to enhance treatment outcomes, particularly in advanced or aggressive cases.

### 3.1. Different Cell Death Pathways Following Radiation

Radiotherapy can induce various forms of cell death, depending on several factors including (1) radiation characteristics such as fraction size, (2) cellular properties such as cell type, cell cycle phase, and antioxidant defences, and (3) the cellular microenvironment, which encompasses tissue oxygen levels [88]. Oxygen levels play a crucial role in radioresistance, with high oxygen levels promoting increased ROS production and thus a better response to radiotherapy. Conversely, low oxygen levels serve as a mechanism of resistance to conventional radiotherapy, leading to decreased ROS generation. Moreover, the presence of oxygen helps to accumulate DNA damage induced by radiation [88,89].

Cell death was previously classified into three types based on morphological features [90]. However, cell death pathways are now defined by the Nomenclature Committee on Cell Death (NCCD), taking into account morphological, biochemical, and functional characteristics [91]. Cell death subtypes are categorised as regulated cell death (RCD), including programmed cell death, and accidental cell death (ACD) [92]. As described by Reindl et al., there are four main types of cell death mechanisms following irradiation: apoptosis, necrosis, autophagy, and mitotic catastrophe [88]. Regarding HNCs, it has been noted that a single dose of 4 Gy administered to various HNC cell lines primarily induces mitotic catastrophe and senescence [93].

Molecular pathways leading to apoptosis, necrosis, and autophagy following radiation are already thoroughly synthesised in other reviews. Interestingly, and as a reminder, mitotic catastrophe is not a distinct form of cell death but rather a cellular stress response that can lead to various other cell death pathways [94,95]. Indeed, it occurs when cells fail to complete proper mitosis [95] or enter mitosis without completing preceding phases [88]. Mitotic catastrophe may result in mitotic arrest, but not always, and cells may die during mitosis, in the subsequent G1 phase, or exit mitosis and enter senescence [88]. Cell death in mitotic catastrophe can also occur during interphase or metaphase following irradiation, and cells may undergo multiple divisions before activating apoptosis or undergoing necrosis [88]. After irradiation, mitotic catastrophe serves as a significant mechanism of cell death. Indeed, X-ray-induced DNA damage disrupts the mitotic cycle, resulting in cellular abnormalities, such as abnormal chromosome segregation and cell division, leading to the formation of multinucleated giant cells and micronuclei [88,94,96]. The fate of cells following DNA damage and mitotic failure depends on the activity of the transcription factor p53. Functional p53 leads to the activation of apoptosis, causing cell death in the subsequent G1 phase. Conversely, in the absence of functional p53, cells continue to divide and accumulate chromosomal anomalies before dying [88]. Additionally, p53 regulates cell cycle checkpoints. In the absence of p53, radiation fails to activate p21, and the CDK2-cyclin A/E complex remains uninhibited. This complex plays a role in centrosome duplication, contributing to the observed hyper-amplification of centrosomes seen in mitotic catastrophe [96]. Notably, HNCs involve additional regulation of p53 in relation to HPV infection, particularly in oropharyngeal cancer, where E6 induces complete degradation of the transcription factor [97].

Other types of cell death have been described after radiotherapy. These include parthanatos, pyroptosis, immunogenic cell death, and senescence [98], and also NETosis and methuosis, which are parts of necrosis along with pyroptosis and ferroptosis [88].

### 3.2. Mechanisms of Ferroptosis Induction After Irradiation

**Figure 3:**
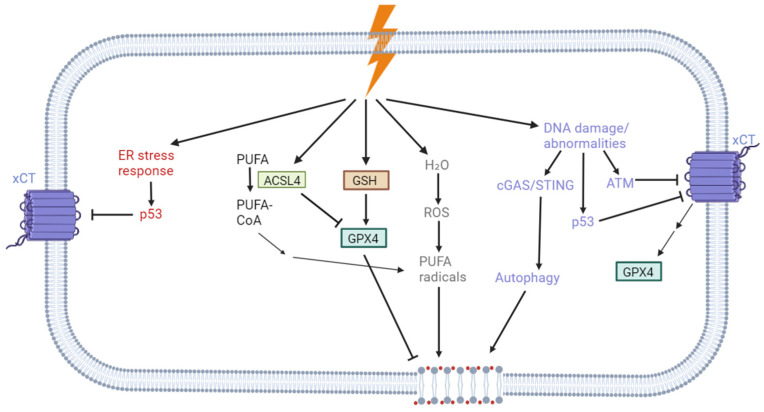
Main pathways identified leading to ferroptosis after radiotherapy, predominantly through lipid peroxidation or the suppression of the antiporter SLC7A11. xCT: cystine/glutamate antiporter, ER stress response: endoplasmic reticulum stress response, p53: tumour protein p53, PUFAs: polyunsaturated fatty acids, ACSL4: acyl-CoA synthetase long-chain family member 4, GSH: glutathione, GPX4: glutathione peroxidase 4, ROS: reactive oxygen species, cGAS: cyclic GMP–AMP synthase, STING: stimulator of interferon genes, ATM: protein kinase ataxia–telangiectasia mutated. Created using BioRender (https://www.biorender.com/).

Ferroptosis is considered a form of regulated necrosis and can also be triggered by ionising radiation (Figure 3) [88]. In 2015, Ivanov et al. demonstrated that administering iron-containing water before radiotherapy enhanced the efficacy of treatment in a rat glioma model [99]. In 2019, Lang et al. revealed that radiotherapy and immunotherapy could induce ferroptosis via the suppression of the subunit SLC7A11 of the Xc^−^ transporter [100]. Their study demonstrated that radiotherapy can induce ferroptosis by increasing lipid peroxidation both in vitro and in vivo. Treatment with a ferroptosis antagonist reduced the effects of radiotherapy. Moreover, ferroptosis agonists were found to sensitise cancer cells to radiotherapy, presenting a novel radiosensitisation strategy [100]. In the same year, Shibata et al. used erastin to radiosensitise adenocarcinoma cells and observed a radiosensitising effect in vitro as well as a decrease in GSH levels and GPX4 protein expression. In vivo experiments showed that pre-treatment with erastin prior to radiation resulted in a significant decrease in tumour volume 15 days after irradiation [101]. Lei et al. also demonstrated that X-ray radiation induced ferroptosis and cell death in different cell lines, including A549, H460, and H1299, which are lung carcinoma models [102].

Thus, several studies have reported that radiotherapy can induce ferroptosis, as evidenced by various markers. Lipid peroxidation has been observed through C11-BODIPY staining or by measuring the levels of specific ferroptosis markers, such as MDA or 4-HNE, along with the observation of morphological alterations of mitochondria. Evidence of ferroptosis induction was also demonstrated by the suppression of radiation effects using iron chelators [94,102,103].

Five pathways have been identified, and these lead to ferroptosis following radiotherapy, predominantly through lipid peroxidation or the suppression of SLC7A11 (Figure 3). These pathways were mainly highlighted in two reviews [104,105].

- DNA damage induced by radiotherapy is recognised by ataxia–telangiectasia mutated (ATM) kinase and associated proteins, implicating the ATM signalling pathway in ferroptosis induction [88] via the inhibition of the SLC7A11 subunit [105]. ATM has been postulated to induce ferroptosis by modulating iron metabolism in response to DNA damage [106]. Activation of the cGAS/STING pathway by DNA abnormalities can also lead to ferroptosis induction, as it can trigger autophagy via STING protein, and this pathway has been shown to promote ferroptosis in specific contexts [105,107].
- Radiation enhances ROS production through water radiolysis, promoting the formation of PUFA radicals (PUFA•) that transform into lipid peroxyl radicals (PUFA-OO•), ultimately resulting in lipid hydroperoxide formation (PUFA-OOH) [104], a key step in ferroptosis.
- Radiotherapy upregulates the ACSL4 enzyme, which catalyses the conversion of PUFAs into PUFA-CoA, which is then esterified via LPCAT3 (lysophosphatidylcholine acyltransferase) [108]. ACSL4 knockout reduces PUFA-containing lipids and then radiotherapy efficacy in vivo [100].
- Radiation exposure depletes GSH levels, reducing GPX4 activity and promoting lipid peroxidation [103,104]. The combination of radiotherapy with ferroptosis inducers synergises for GSH depletion [103], likely due to SLC7A11 inhibition, leading to decreased cysteine levels and GSH synthesis [100].
- The activation of p53 by radiotherapy contributes to SLC7A11 suppression [109] through two pathways: (1) activation of the ER stress response via the sensor protein PERK, leading to p53 activation [105,110], and (2) p53 activation via the DNA damage response signalling cascade [74,81,82,109,111]. Subsequently, p53 represses *SLC7A11* transcription, reducing GSH levels and GPX4 activity [98]. It is important to note the ambiguous role of p53 in ferroptosis. While p53 can influence various targets within the ferroptosis pathway, its precise role as a tumour suppressor in this process remains controversial. Although many studies suggest that p53 promotes ferroptosis, its impact varies depending on contextual factors such as the specific cell lines under study, the levels of stress factors, and inherent gene expression profiles. These variations lead to differential signalling pathways upon p53 activation [111,112].

### 3.3. Limitations in Radiotherapy Treatment of HNCs

As stated in the introduction, radiotherapy is an essential part of the treatment of HNCs. It may be used alone as an alternative to surgery in stage I and II diseases or in combination with cisplatin, either as a radical treatment or after surgery for locally advanced HNCs. Radiotherapy itself has undergone a revolution in the last thirty years with the introduction of intensity-modulated radiotherapy (IMRT) in combination with image-guided radiotherapy (IGRT), allowing for more precise targeting of tumours while better sparing radiosensitive healthy tissues surrounding the tumours. Additionally, modifications to radiotherapy treatment regimens, such as (I) hyperfractionation, which allows healthy tissues to recover between treatment fractions, thereby limiting toxicity, and (II) accelerated radiotherapy, designed to reduce cancer cell recovery, have contributed to improved tumour control.

In the same period, concomitant treatment with cisplatin and irradiation became a standard of care in non-operable locally advanced head and neck cancers (LAHNCs), adding 6.5% to the five-year survival [113]. In the adjuvant setting, cisplatin is indicated in the case of positive margins and capsule rupture [114,115,116]. However, despite the advances in radiotherapy, the toxicity of this combination therapy remains high [117] and the prognosis is reserved (5-year survival of 40–60%). Attempts to replace cisplatin with a less toxic radiosensitiser have largely failed. In this context, the monoclonal anti-EGFR antibody Cetuximab (a targeted therapy), the most promising of them all, was for a long time a recommended alternative for cisplatin-unfit patients after an initial publication in 2006 [118] about its superiority over radiotherapy alone. However, it finally failed to show an advantage over classical cisplatin in a randomised way [119,120]. Not only was patient survival lower, but the toxicity profile was also less favourable. Finally, the very popular and often-efficient immunotherapy has so far failed to show an added beneficial value in non-metastatic HNCs [121,122].

Related to toxicity, efficacy is an even bigger problem, even in HPV-positive HNCs known to have a better prognosis than the classical alcohol–tobacco-related HPV-negative HNCs. Despite one of the highest doses used in the field of radiotherapy (i.e., 70 Gy), HNCs most often recur within the high-dose area, due to intrinsic resistance and the hypoxic nature of these cancers [123,124]. Duprez et al. showed the dose-limiting toxicity to be mucosal ulcers, limiting the dose at 84 Gy [125,126].

The combination of high toxicity and suboptimal efficacy in current state-of-the-art concomitant radiochemotherapy demands urgent research into alternative solutions. As radiotherapy schemes appear to have reached their maximum tolerated doses and systemic radiosensitisation remains largely dependent on the outdated yet toxic cisplatin, new options are essential. In this context, we emphasise our contributions by focussing on the radiosensitising potential of IONPs as a promising and novel approach. Our review reports and explores their ability to enhance radiotherapy efficacy while potentially minimising toxicity, offering a significant advancement in the search for safer and more effective radiosensitisation strategies.

Thus, radiotherapy remains a key treatment of HNCs, serving as a primary or combination therapy to improve patient outcomes. However, limitations due to toxicity, hypoxia-induced radioresistance, and suboptimal efficacy underscore the need for innovative radiosensitisation strategies to improve treatment while minimising adverse effects, by using IONPs, for example.

## 4. Iron Oxide Nanoparticles (IONPs)

IONPs have emerged as a promising strategy to enhance the efficacy of radiotherapy in cancers. Their unique properties, such as efficient radiation dose enhancement, targeted delivery, and minimal toxicity, make them ideal candidates for improving tumour radiosensitivity while reducing damage to surrounding healthy tissues. By exploiting their ability to generate ROS and therefore amplify local radiation effects, IONPs could represent a significant advancement in overcoming current limitations associated with radiotherapy.

### 4.1. Structural Descriptions of the IONPs

**Figure 4:**
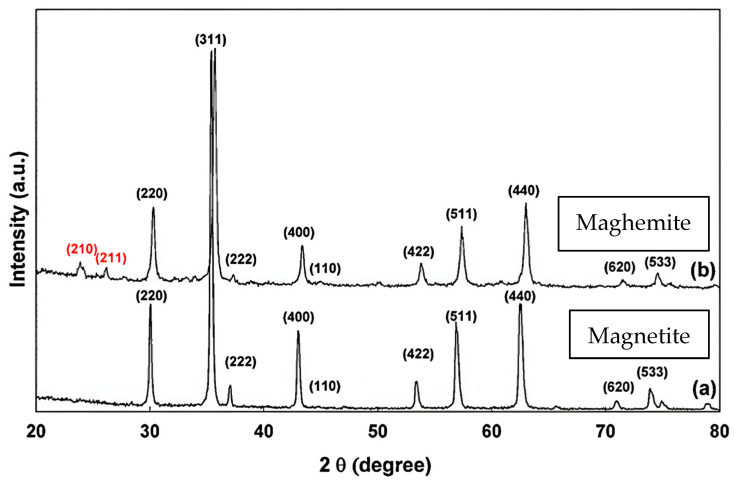
Comparison of the XRD spectra of maghemite (**a**) and magnetite (**b**). Maghemite is known to present two additional peaks (in red; 210 and 211) in comparison to the magnetite. Reprinted with permission from [127].

Among the nanomaterials studied for biomedical applications, IONPs have become a keystone through the years due to their high surface–volume ratio, biocompatibility, and magnetic properties [33]. These nano-objects are characterised by a magnetic core ranging from 1 to 100 nm in diameter organised in a crystalline reverse-spinel structure. Among the IONPs studied in a biomedical context, maghemite (γ-Fe_2_O_3_) and magnetite (Fe_3_O_4_) appear to be the most frequently used. Both iron oxide phases are organised in a face-centred cubic lattice structure. They differ, however, in the organisation of iron ions and oxygen ions in the mesh. In the case of magnetite, the lattice is made up of 32 oxygen anions and 24 iron cations, which are distributed between 8 tetrahedral and 16 octahedral positions. In the case of maghemite, divalent iron ions are absent in the crystalline structure. As a result, trivalent iron ions are distributed between the tetrahedral and octahedral sites, implying a deficiency of iron ions in the octahedral sites. These structural differences can be reflected in X-ray diffractograms (XRDs). Maghemite has two diffraction peaks (Miller index = (210) and (211)), which are absent from the magnetite XRD (Figure 4).

For both systems, the structural arrangement of iron ions and oxygen within the crystal, in combination with their size/shape give the nanomaterial unique magnetic properties (i.e., superparamagnetism and high magnetic susceptibility) [33]. Their magnetic properties have been widely used for biomedical applications, such as contrast agents for MRI [128], targeted magnetic delivery [129], induction of local magnetic hyperthermia or magnetically induced cell lysis [130], and more recently, magnetic particle imaging (MPI) [33]. In addition, their surface can be easily modified by stable organic coatings to allow further functionalisation [131,132,133], increased colloidal stability [129,134,135], exogen material delivery [133,135], or increased in vivo circulation time [136]. The latter point is of the utmost importance, as several studies have shown that clearance from the bloodstream and biodegradation of IONPs occur mainly by filtration in the kidneys or by capture in the liver and spleen, despite many variations through the physicochemical properties of IONPs. A longer circulation time is therefore associated with a greater probability of reaching the area of interest [137].

Another example of IONP surface modification involves multimodal imaging applications. For example, Stanicki et al. demonstrated the possibility of using IONPs for in vivo trimodal imaging (including MRI, fluorescence imaging, and multispectral optoacoustic tomography (MSOT)) through the grafting of ZW-800 (a near-infrared fluorophore) to the surface of IONPs [136]. Another example of multimodal imaging related to IONPs was given by Xie et al. who designed a platform of human serum albumin-coated IONPs dually labelled with (64)Cu-DOTA and Cy5.5 suitable for in vivo positron emission tomography (PET)/fluorescence and MRI tri-modality imaging [138].

**Figure 5:**
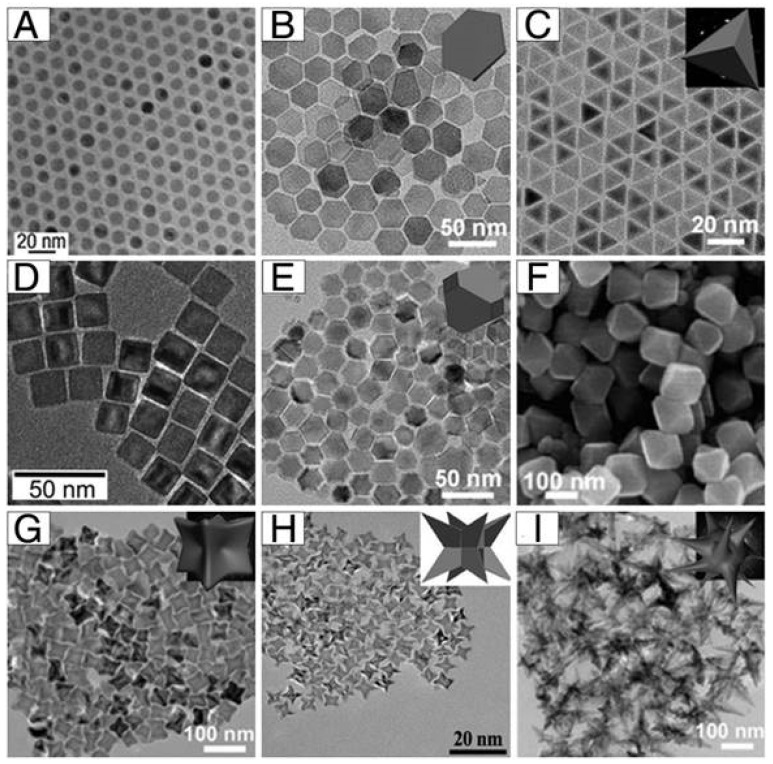
Representative transmission electron microscopy (TEM) images of variously shaped IONPs. (**A**) Nanospheres, (**B**) hexagons, (**C**) tetrahedrons, (**D**) cubes, (**E**) truncated octahedrons, (**F**) octahedrons, (**G**) concave cubes, (**H**) octapods, and (**I**) polypods. Reproduced from [139] under the terms of the Creative Commons Attribution (CC BY-NC) license. Since the potential applications of an IONP platform depend on its physicochemical properties (i.e., size, surface–volume ratio, crystallinity, and shape) (Figure 5), which can be modified through the synthesis processes employed, numerous synthesis strategies have been developed and optimised throughout the years. Each of them has their advantages and their disadvantages, which are briefly summarised in Table 2.

In addition to the few examples given below, many other remarkable studies have been published in the literature concerning this very particular aspect of IONPs synthesis. However, since the parameters affecting the size, shape, and composition of nanoparticles (Figure 5) are closely interconnected and interdependent, it remains challenging to fully comprehend the process or accurately predict how a specific parameter impacts the final synthesised NPs.

**Table 2:** Overview of the advantages and limitations of the main IONPs synthesis strategies.

Method	Advantages	Disadvantages	Shape	References
Coprecipitation in aqueous media (also known as Massart’s method)	High yield Direct production of water-dispersible particles	Low temperature (below 100 °C) Poor size control Low crystallinity	Amorphous	[13,136,140,141,142,143,144]
Polyol process	High yield Direct production of water-dispersible particles High temperature	Poor size control Medium crystallinity	Spheres Amorphous	[145]
Micro-emulsion strategy	High size control High crystallinity	Low yield	Spheres Cubes	[146,147,148]
Sol–gel method	High size control High crystallinity	Low yield	Spheres	[149,150]
Thermo-decomposition	High size control High crystallinity	Medium yield	Hexagons Tetrahedrons Octahedrons Octapods Concave cubes Multiple branches	[151,152,153,154]

In addition to the few examples given below, many other remarkable studies have been published in the literature concerning this very particular aspect of IONPs synthesis. However, since the parameters affecting the size, shape, and composition of nanoparticles (NPs) are closely interconnected and interdependent, it remains challenging to fully comprehend the process or accurately predict how a specific parameter impacts the final synthesised NPs.

A complete review of this topic is out of the scope of this review. Nevertheless, it is crucial to explore how these technological advancements translate into practical applications, particularly in the medical field. Among the many areas of investigation, oncology stands out as one of the most promising, especially in the treatment and diagnosis of complex cancers such as those of the head and neck. These conditions, marked by their heterogeneity and therapeutic challenges, directly benefit from innovations involving IONPs. In this chapter, we will delve into recent research highlighting the potential of IONPs to revolutionise current approaches in early detection, targeted drug delivery, and new therapies for HNCs. To this end, the following section will provide an overview of recent research on such cancers involving the use of IONPs.

### 4.2. Application of Drug and Gene Delivery Strategies

Several studies focus on the possibility of using IONPs as vectors for drug delivery due to their high surface–volume ratio. The major advantage of using such vectors is their ability to potentially improve the poor pharmacokinetic properties of some drugs (poor solubility, high systemic toxicity, non-specific delivery, and short circulating half-lives), by binding these agents to IONPs [155] (Table 3). For example, cisplatin is one of the main first-line treatments considered for various types of cancer, but its use is widely limited due to numerous adverse events [156]. With a view to countering this limitation, Bejjanki et al. studied the potential of cisplatin-loaded IONPs coated with folic acid [133]. This study showed that such a formulation was able to induce higher ROS production compared to non-vehicle cisplatin in a nasopharyngeal carcinoma cell line (HNE-1), with limited off-target toxicity. Weng et al. have observed similar therapeutic outcomes following the evaluation of IONPs modified with the transactivator of transcription-derived (TAT; GRKKRRQRRRPQ) peptide and encapsulating cisplatin in HNE-1 and CNE-2 cell lines [157].

On a different note, Zhang et al. studied a new formulation of mesoporous IONPs functionalised with bleomycin, a cytotoxic antibiotic commonly used in the context of HNSCC treatment, on both in vitro and in vivo HNC models [158]. Their results indicated a better accumulation of bleomycin in the pathological region when the drug is carried by IONPs compared to unbound bleomycin. The as-described system was also capable of inhibiting the growth of tumours in vivo.

In addition to small drugs, IONPs have also been described as carriers for biological molecules with therapeutic aims. For instance, Miao et al. described the use of IONPs as gene transfer vectors for tumour necrosis factor-related apoptosis-inducing ligands in order to induce strong apoptotic behaviour in a model of oral squamous cell carcinoma (OSCC) in the presence of a magnetic field [159]. Alternatively, Jing et al. assessed the effect of ribonucleic acid (miR-504)-loaded IONPs on a cell line derived from a squamous cell carcinoma of the tongue of a 25-year-old patient (namely, the SCC-9 cell line) [160]. This study showed that IONP-mediated transfection of miR-504 results in a loss of cyclin-dependent kinase 6 and consequently inhibits oral cancer cell migration through modulation of the ERK signalling pathway.

A global summary of these studies shows that IONPs can be considered as promising nanotransporters in the fight against HNCs for various types of molecules including metal chemotherapeutic complexes (i.e., cisplatin), cytotoxic organic molecules (i.e., bleomycin), or apoptosis-inducing cytokine. On the one hand, numerous studies seem to show a preferential accumulation of the active molecules in cancer cells when associated with IONPs. This preferential accumulation has been described both in the presence and in the absence of a guiding magnetic field.

On the other hand, the degradation products of IONPs seem in some cases to have a synergistic effect with the action of the active molecules carried by IONPs, as described in the work of Weng et al., in which the combined action of cisplatin and iron ions seems to lead to a loss of resistance of cells to cisplatin [157]. It should be noted, however, that relatively few studies to our knowledge have looked at the use of IONPs for drug delivery in the context of HNC applications. This area could benefit from further study.

### 4.3. Targeting Cancer Cells with IONPs

Following the descriptions of the various strategies used by IONPs in the context of HNCs, the effectiveness of these approaches remains linked to the accumulation of IONPs within the pathological zone and not in healthy tissue. A simplistic view of the phenomena leading to the accumulation of IONPs in the tumour is based on passive targeting mechanisms resulting from the enhanced permeation and retention (EPR) effect [161]. This effect, first theorised by Maeda in 1986 [162], relies on several features displayed in the tumour zone, including highly permeable vasculature promoting the enhanced permeability of particles and impaired lymphatic drainage promoting their enhanced retention. However, this EPR effect has shown little relevance to human cancers in clinical studies compared with preclinical studies on animal models [161]. Given the lack of effectiveness of the EPR effect in clinical trials, there are little to no relevant reports on the use of IONPs in the context of HNCs. Instead, different targeting strategies have been developed.

One that can be implemented to promote the accumulation of IONPs within the tumour is based on the intratumoural injection of nanoparticles. Numerous studies have shown that this type of injection produces greater intratumoural accumulation compared to the intravenous injection of nanoparticles [163,164,165,166]. However, this strategy can quickly be limited in its realisation, mainly by the tumour’s lack of accessibility for intratumoural injection.

Consequently, the development of new strategies involving biological targeting has been the subject of much interest. More specifically, targeting ligand (TL) grafting is a strategy which is widely documented in the literature. TLs usually take the form of small organic molecules [133], peptides [132,157,167], aptamers [168], or antibodies [130]. From a mechanistic point of view, this strategy implies the introduction of the TL onto the surface of IONPs, and this TL exhibits a high affinity for molecules specifically exposed on the surface of tumour cells. The interaction between these two molecules is then supposed to promote the internalisation of targeted IONPs.

However, this goal can be widely limited by the heterogeneity and variations in receptor expression across different tumour zones and at different tumour stages. To illustrate this issue, several studies have shown that the expression of EGFR can be widely variable over time or depending on the location of cancer cells within a tumour or whether the tumour area is primary or metastatic [169,170,171,172]. In the light of these findings, the future of TL-mediated delivery strategies for IONPs against cancer cells will require a more detailed understanding of TL target expression variations in a cancer context.

In addition, ensuring stability in physiological conditions while retaining binding affinity appears to be a critical point for efficient targeting against cancer cells. To this end, researchers have explored various optimisation strategies including surface modifications by organic polymers such as PEG for minimising protein corona formation and preventing aggregation. These methods ensure that the targeting ligands retain their binding affinity while operating in complex biological systems [173,174]. Also, optimisation of TL conjugation techniques, such as favouring the covalent binding of targeting ligands to the nanoparticle surface over physical adsorption, could enhance stability, targeting specificity, and therapeutic efficacy, as well as ensure a stable attachment under physiological conditions, reducing the risk of ligand detachment or loss of targeting functionality [174].

In addition to these considerations, the conjugation pattern (i.e., the number and the degree of clusterisation) of a TL on a nano-object has also been shown to be a key factor in an effective vectorisation strategy. Indeed, Fang et al. showed that 9 nm sized miniferritin protein nanocages functionalised with 2 or 12 copies of RGD peptide showed better delivery than similar nano-objects functionalised with 1 or 24 copies of RGD peptide [175]. This case highlights the importance of both chemical and biological considerations when designing an efficient vectorisation strategy.

In the light of a study proposed by Balk et al., who investigated the impact of an external magnetic field (380 mT) on the internalisation of lauric acid- and human serum albumin-coated IONPs in vitro in HNC cell lines, magnetic-beam-mediated vectorisation appears as an alternative promising guidance strategy [129]. Specifically, Balk et al. showed that the application of an external magnetic field significantly increased the quantity of IONPs internalised in the different cell lines [129]. In the context of this study, magnetic guidance to the pathological area could be considered a promising strategy to exploit the magnetic properties of IONPs for targeted delivery.

### 4.4. Induction of Ferroptosis by IONPs and Other Nanoparticles

The previous parts of this work have already extensively described the known mechanisms responsible for ferroptosis phenomena. Given the close relationship between intracellular iron accumulation and ferroptosis, numerous studies have considered the possibility of using IONPs to induce ferroptosis in cancer cells [176,177,178,179,180].

As an example, the work of Fernández-Acosta et al. exposed multiple cancer cell lines to gallic acid- and polyacrylic acid-functionalised IONPs, resulting in the induction of cell death [179]. However, this phenomenon was reduced when cells were previously exposed to a ferroptosis inhibitor. Similar results were revealed by Lomphithak’s team using PEGylated IONPs [177].

By creating a hybrid nanoparticle with a biocompatible oleic acid-coated Fe_3_O_4_ core, PSN peptide, oxaliplatin, and Prominin2siRNA, Wang et al. presented a trifunctional IONP platform [181]. More specifically, siProminin2 acts as an inhibitor of the exosomal process while the PSN peptide acts as a targeting vector against tumour cells. Additionally, the iron oxide core was able to induce both a ferroptotic process and immunogenic cell death. It appeared that these effects were significantly modulated through the action of oxaliplatin on a murine orthotopic breast cancer model.

However, despite all the reported examples of the use of IONPs for ferroptosis induction strategies, there is a large body of research on the use of non-iron oxide nanoparticles for ferroptosis induction in cancer cells. A good example is reported by Zhang et al., who studied the possibility of using nanoparticles [182] composed of human serum albumin core onto which ferric porphyrin, celecoxib, and roscovitine were grafted. The advantage of this platform is its ability to form a multimodal nanoparticle combining multiple roles. Indeed, the ferric porphyrin acts as an iron carrier, the content of which is involved in the Fenton reaction to produce ROS. Secondly, Celecoxib reduces inflammation-associated immunosuppression by inhibiting the inflammation-related COX-2/PGE2 pathway, which is activated by ferroptosis, whereas roscovitine suppresses the Cdk5 pathway to stop IFNγ-dependent *PD-L1* gene transcription and minimise adaptive immune resistance through the genetic blockade effect. Finally, human serum albumin acted as a central anchor for the formation of the nanostructure through hydrophobic interaction but was also involved in the favoured accumulation of the nanostructure in the tumour due to the overexpression of albumin-binding protein on the membrane of cancer cells. The as-prepared formulation was shown to induce ferroptotic damage and immune responses that act in a synergistic way against the survival of cancer cells. Moreover, a GPX4 pathway disruption was noticed and led to a cascade amplification of ferroptotic cell mortality and ferroptosis-induced immunotherapeutic effectiveness. Specifically, these nanoparticles effectively addressed the intrinsic drawbacks of ferroptosis in immunotherapy by removing inflammation-associated immunodeficiency and reversing interferon-γ adaptive immune resistance to maximise the effectiveness of immunotherapy due to the combination of roscovitine and celecoxib in the nanostructure. Another study conducted by Han et al. focussed on the synthesis and evaluation of a pyrophosphate core nanoconstruct in order to intracellularly deliver a cholesterol-like derivative of dihydroartemisinin and pyropheophorbide–iron [183]. This study concluded that the intracellular delivery of these two components was able to increase ROS production in tumour cells, favour the immunogenic response against the tumour, and inhibit tumour growth in a murine model of colorectal cancer.

In another study, Li et al. showed that it was possible to induce ferroptotic behaviour without modulations on intra- or extracellular iron but directly through modulation of the lipid peroxidation process. They described a novel nanoplatform made of glycyrrhetinic acid (a pentacyclic triterpenoid) able to inhibit the expression of glutathione-dependent peroxidases 4 (GPX4) and induce a ferroptotic process in leukaemia and colorectal cancer without modulation of the intracellular iron pool [184].

Additionally, Song et al. have developed intracellular-acid-activable dynamic nanoparticles for the tumour-targeted delivery of RSL-3, a ferroptosis inducer acting as a GPX4 inhibitor [185]. The nanoparticles were engineered through an assembly of poly (ethylene glycol)-block-poly(2-(diisopropylamino)ethyl methacrylate) diblock copolymers and acid-liable phenylboronate esters, which can sequestrate RSL-3 inside the hydrophobic core via π–π stacking interactions. An in vivo evaluation of this model led to the induction of ferroptotic processes and increased immunogenicity against the tumour.

Hence, IONPs possess unique structural and functional properties, such as their high surface–volume ratio, biocompatibility, and magnetic characteristics, making them useful platforms for biomedical applications, particularly in oncology. Their ability to enhance drug delivery, facilitate targeted therapies, and induce ferroptosis highlights their potential to revolutionise treatment strategies for complex cancers like HNCs.

## 5. Combining Radiotherapy and IONPs for Sensitisation Through Ferroptosis

As seen in the previous section, IONPs hold great promises in the field of cancer therapy. In this new section, we would like to dive into the combination of metallic nanoparticles with radiotherapy. Indeed, such a strategy has emerged as a promising combination in oncology, particularly for exploiting the radiosensitising effects of nanoparticles. As explained in the comprehensive review of S. Penninckx et al., metallic nanoparticles exhibit a dual mechanism for enhancing radiotherapy outcomes. Firstly, they amplify radiation effects by increasing the deposited dose through the emission of secondary X-rays or Auger electrons upon radiation exposure. Secondly, metallic nanoparticles exert radiosensitising effects by modulating the ROS balance within cells. This modulation occurs through two key mechanisms: (1) the promotion of ROS production via water radiolysis and (2) the suppression of antioxidant defences through the inhibition of various antioxidant enzymes [186,187].

More recently, Jordan Da Silva et al. showed another pathway by which metallic nanoparticles can execute their activities [188]. An investigation of hafnium oxide-containing nanoparticles, symbolised by NBTXR3, has unveiled novel insights into their radiosensitising properties. Mechanistic studies have revealed that the combination of radiotherapy and NBTXR3 caused an increase in lysosomal membrane permeabilisation where there was none observed in radiotherapy alone (Table 4). In addition, the combination induced a significant increase in lipid peroxidation and then ferroptosis compared to radiotherapy alone. The authors suggested that those two phenomena might explain the immunogenic cell death observed with the combination and highlighted that radiotherapy combined with NBTXR3 acted on multiple pathways and not only enhanced the effects of radiotherapy. Clinical trials using NBTXR3 for locally advanced soft tissue sarcoma and head and neck squamous cell carcinoma have already demonstrated their efficacy in increasing radiotherapy outcomes and presenting a good safety profile, respectively (NCT02379845, https://clinicaltrials.gov/, accessed on 2 September 2024). It is important to note that the effects of combining radiotherapy and metallic nanoparticles, including IONPs, are also partially explained by the effects on the microenvironment. Indeed, metallic nanoparticles can switch the phenotype of M2 (protumoural) macrophages to M1 (antitumoural) as well as promote immune cell recruitment at the tumour site [189].

Gadolinium nanoparticles are also being studied for their potential in sensitising tumours to radiotherapy and inducing ferroptosis. These nanoparticles are currently in clinical trials for phase I and/or phase II studies in combination with radiotherapy for various cancers including brain metastases. Hao Sun and Hui Cai et al. investigated their effects in models of triple-negative breast cancers. They showed that gadolinium-based nanoparticles and radiotherapy were able to induce more ferroptosis than the other group tested. These effects were characterised by alterations in mitochondrial morphology, increased lipid peroxidation, and elevated levels of 4-hydroxynonenal (4-HNE), the peroxidation product. Moreover, they highlighted an inhibition of the pathway NRF2-GSH-GPX4 after irradiation combined with nanoparticles and it could constitute the mechanism for radiosensitisation [190].

IONPs have also exhibited radiosensitising properties in a model of lung carcinoma, with exposure 24 and 48 h before irradiation, employing two distinct IONP coatings. Interestingly, these effects appeared unrelated to the iron content after IONP incubation. Ternad et al. showed a decrease in the activity of the thioredoxin reductase enzyme with both formulations of IONPs [13]. Thioredoxin reductase plays a pivotal role in cellular redox homeostasis by catalysing the reduction in thioredoxin, a key regulator of cellular processes involved in oxidative stress response and cell survival. Consistent with previous findings by Penninckx et al., which highlighted the ability of gold nanoparticles to inhibit the activity of this enzyme and contribute to the radiosensitising effect of gold nanoparticles [191,192], it was further elucidated that iron ions themselves can modulate the activity of thioredoxin reductase through the oxidation of critical groups within its active site, such as thiol and selenol groups [13].

In 2022, Yingbo Li and Jie Yang et al. investigated the effects of iron oxide nanoclusters (i.e., nanoplatforms composed of multiple magnetic cores resulting from a controlled aggregation), sensitive to pH for iron release, in a model of lung cancer via pulmonary delivery. They observed an increase in apoptosis and ferroptosis induction with the combination of nanoparticles and radiotherapy in vitro, along with a decrease in tumour growth in vivo [193].

Furthermore, Chaewon Bae et al. developed a nanoparticle model based on iron and hyaluronic acid, which was demonstrated to induce ferroptosis [194]. This nanoparticle model was subsequently utilised in combination with radiotherapy in both in vitro and in vivo studies by the same research group [197]. Their findings revealed that the combination treatment enhanced cancer cell killing while simultaneously increasing lipid droplet formation within exposed cells. This observation suggests an additional pathway linking ferroptosis induction to radiosensitisation, with lipid droplets acting as facilitators of ferroptosis under external stressors (such as iron or radiotherapy). This mechanism drives cancer cell death through ferroptotic pathways. On the other hand, a recent study found that lipid droplet formation following cell cycle arrest could contribute to resistance to ferroptosis [198]. Specifically, cancer cells that exhibited resistance to chemotherapy (5-FU) or radiation and displayed a slow-cycling phenotype showed increased lipid droplet accumulation, which correlated with ferroptosis resistance. This resistance is likely due to the sequestration of PUFAs within lipid droplets, thereby preventing them from participating in the lipid peroxidation necessary to trigger ferroptosis. These findings highlight the complexity of ferroptosis pathways in the context of treatment resistance and underscore the importance of exploring ferroptosis mechanisms to inform novel therapeutic strategies involving ferroptosis inducers. Interestingly, while numerous studies have shown that ferroptosis inducers can enhance radiotherapy effects, the effects of radiotherapy itself often involve cell cycle arrest, which can suppress ferroptosis. Dixon and Olzmann emphasised that the role of lipid droplets in ferroptosis sensitivity is context-dependent, particularly with resistance mechanisms and PUFA sequestration [43].

Ying-Ke Hou and Zi-Jian Zhang et al. studied another very ingenious iron-containing nanoplatform for the treatment of breast cancer. The core of the nanoplatform is composed of Fe^3+^ and polydopamine, and the shell is composed of platinum, which is then covered by hyaluronic acid. Each component has an anti-cancer effect. The ferric iron present can deplete GSH and promote hydroxyl radical production, both of which promote ferroptosis. The combination with X-rays induced a decrease in viability as well as an increase in ROS levels in vitro and a significant decrease in tumour volume in vivo. The combination also showed a significant decrease in GSH in the presence of light (photothermal therapy) [195].

Another recent study investigated metal oxide nanocomplexes (ferrous oxide or copper oxide) coupled with diethyldithiocarbamate (DE) in models of human and mouse stem cell glioblastoma and their radiotherapy-resistant counterparts. Ferrous oxide nanoparticles gave the best results for growth inhibition in both sensitive and radiotherapy-resistant cells compared to temozolomide (chemotherapy) and copper oxide nanocomplexes. The ferrous oxide nanoplatform was also able to sensitise the different cell lines tested to temozolomide and to radiotherapy, at different concentrations regarding the cell line tested. This is explained by the inhibition of ALDH1A1 by DE and the induction of ferroptosis promoted by DE and ferrous ions [196].

Lastly, Haonan Tang et al. studied a nanoplatform composed of superparamagnetic IONPs, erastin, and polyethylene glycol in the context of nasopharyngeal cancer. This platform aims to be a theranostic tool by helping in the monitoring of the disease through its MRI contrast agent property and a therapeutic tool by inducing ferroptosis. Indeed, the platform showed better antitumour activity in vitro and in vivo. Plus, the platform seems to have increased the solubility of erastin [199]. The study did not evaluate the effects of the combination with radiotherapy but showed an interesting model by directly coupling a strong ferroptosis inducer to the nanoparticle model.

The induction of ferroptosis requires several factors, such as the presence of elevated ferrous iron, ROS imbalance, and lipid peroxidation. Regarding HNCs, a study showed an increase in ROS generation when cells were exposed to IONPs [133]. Another group also showed the radiosensitising potential of IONPs on HNCs [134]. Moreover, in the “Ferroptosis” section, we discussed several studies that targeted ROS production directly or antioxidant defences to induce ferroptosis in HNC cells, and we also discussed the pathways by which radiotherapy can induce ferroptosis.

Based on these interesting results, we hypothesise that the use of IONPs combined with radiotherapy in HNC cells can increase radiotherapy outcomes, notably through the elevated ROS generation and the induction of ferroptosis. To date, there is still a lack of studies investigating this specific topic. However, the available data lead us to think that IONPs, through their iron content, could generate elevated intracellular ferrous iron and impact antioxidant defences such as the TrxR enzyme. This approach is specifically interesting in HNCs as targets from the ferroptotic pathways are associated with a poor prognosis, which make them vulnerable to this treatment. The subsequent ROS imbalance, combined with radiotherapy effects, could lead to increased cell death and increased ferroptosis.

Given the pressing need for novel radiosensitising agents in the management of HNCs, coupled with the potential benefits of ferroptosis induction, exploring this avenue holds significant promise. Locoregional relapse within five years post-treatment affects approximately one-third of patients, posing substantial morbidity and mortality risks due to potential disability and pain [200]. As described in a previous section, radioresistance in HNCs can arise from different factors such as an increased ability of cancer cells to repair DNA damage [200] as well as a higher density of cancer stem cells (CSCs) [201]. Interestingly, inducing ferroptosis could be a new way of targeting these CSCs and reverse radioresistance. Indeed, CSCs seem to be more sensitive to ferroptosis than to other types of cell death and rely on iron metabolism to support their function and proliferation [202].

Several studies have developed nanoparticle models capable of specifically targeting CSCs and rendering them vulnerable through the induction of ferroptosis. For instance, Zhao et al. conjugated salinomycin to gold nanoparticles coated with PEG and demonstrated ferroptosis induction, which significantly enhanced the sensitivity of breast cancer stem cells to this formulation [203]. Another study investigated the therapeutic effects of superparamagnetic iron oxide nanoparticles (SPIONs) coupled with atranorin, a secondary metabolite produced by certain lichens, on CD44⁺ gastric CSCs. These nanomaterials effectively sensitised CSCs by modulating several ferroptosis-related targets, such as Xc⁻ transporters and GPX4. This induction of ferroptosis resulted in decreased viability and translational activity of CSCs [204]. Recently, nanocomplexes of diethyldithiocarbamate (DE), an inhibitor of aldehyde dehydrogenase 1A1 (ALDH1A1), conjugated with ferrous oxide nanoparticles (FeO NPs), demonstrated remarkable efficacy by inhibiting the growth, chemoresistance, and radioresistance of glioblastoma CSCs. These potent anti-CSC effects were primarily attributed to the downregulation of anti-ferroptosis factors, such as ALDH1A1 activity, GSH levels, and GPX4 activity, thereby enhancing DE-FeO NP-induced ferroptosis [196]. Additionally, Chittineedi et al. highlighted the critical role of ferroptosis in targeting breast CSCs using gold nanoparticles conjugated with polyherbal formulations [205]. Another innovative approach targeting CSC clusters in triple-negative breast cancers involved EGFR receptor blockade to sensitise CSCs to ferroptosis. Researchers developed ferritin-based nanoparticles loaded with lapatinib, an EGFR inhibitor, and pseudolaric acid B (PAB), a ferroptosis inducer. This strategy significantly reduced spheroid formation and increased ROS production via ferroptosis [206]. Consequently, numerous emerging approaches involving customizable and versatile nano-objects have demonstrated direct effects on ferroptosis induction. These promising anti-cancer therapies targeting CSCs warrant further investigation to enhance their efficacy, tumour retention, and specificity in future applications.

Moreover, as described above, strategies developed to induce ferroptosis in HNCs showed promising outcomes in countering chemo- and radioresistance. More recently, Yuting Chen et al. demonstrated ferroptosis induction after irradiation in models of nasopharyngeal carcinomas and showed the induction of protein glutathione S-transferase mu 3 (GSTM3) expression after irradiation [207]. Interestingly, GSTM3 was found to confer radiosensitivity to nasopharyngeal carcinoma cells in vitro by inhibiting the expression of GPX4 and influencing the production of PUFAs by indirectly increasing polyunsaturated fatty acid production via fatty acid synthase (FASN) enzyme stability. The authors suggest that these pathways lead to ferroptosis induction and radiosensitivity in nasopharyngeal carcinomas. Notably, FASN enzyme production has been implicated in inducing radioresistance in nasopharyngeal carcinomas in previous studies, further highlighting the intricate interplay between ferroptosis regulation and radioresistance mechanisms in HNCs [208,209].

Therefore, combining metallic nanoparticles like IONPs with radiotherapy represents a promising strategy for enhancing cancer treatment by exploiting their radiosensitising effects and their ability to induce ferroptosis. This dual mechanism offers potential pathways to overcome radioresistance, target cancer stem cells, and improve therapeutic outcomes, warranting further investigation into their clinical application.

## 6. Perspectives

The development of advanced nanoparticle systems and ferroptosis-inducing strategies offers promising avenues for improving cancer therapy, particularly in overcoming treatment resistance and enhancing radiosensitivity. However, translating these findings into clinical practice remains a challenge, requiring further validation through advanced in vivo models such as organoids and patient-derived xenografts (PDXs).

Despite the promising advances mentioned above and the progress in nanoparticle system design, further improvements are necessary to bridge the gap between in vitro findings and the integration of these models into clinical practice. The increasing adoption of organoid models and PDXs enhances the relevance of these nano-objects as anti-cancer therapeutic tools. Indeed, using various sophisticated formulations, several studies have successfully translated and validated their ferroptosis-inducing effects in PDX mouse models of different cancers. Tumour tissues from treated PDX models have notably shown reduced GPX4 expression [210,211].

Similarly, the use of patient-derived organoids and xenograft models has confirmed the role of the SLC7A9 transporter in gastric cancer progression through its inhibitory effect on ferroptosis. Knockdown of *SLC7A9* in these in vivo models demonstrated a stronger induction of ferroptosis by erastin compared to the control groups. Likewise, a study on mucosal melanoma overcame resistance mechanisms to ferroptosis and showed tumour growth inhibition by combining EZH2 inhibition with erastin, a ferroptosis inducer [212].

In the future, it will be essential to continue integrating advanced models, such as patient-derived organoids and PDXs, to validate hypotheses through proof-of-concept studies. This approach holds great promise for accelerating the translation of these innovative treatments into clinical trials.

Besides the progress of new experimental models, innovative nano-objects are also being developed to improve cancer treatment. A recent study proposed a nanocomposite combining metal–organic frameworks (MOFs) with magnetic nanoparticles to enhance drug delivery for cancer treatment. MOFs, known for their high surface area, tuneable pore size, and biocompatibility, are surface-modified with polymers to improve stability, solubility, and targeting capabilities, reducing toxicity. When combined with magnetic nanoparticles like manganese ferrite (MnFe_2_O_4_), the nanocomposite leverages ROS generation and hyperthermia for tumour destruction, improving drug delivery in cancer therapy [213]. Also, in this context, Tiwari et al. reviewed the growing interest in single- and dual-atom catalysts, also known as nanozymes, which exhibit superior catalytic activity compared to conventional nanoparticles [214]. These nanozymes, mimicking natural enzymes, are particularly valuable in cancer therapy due to their ability to generate ROS and induce ferroptosis. Iron-based nanozymes, such as FeN3P, show promising potential in tumour suppression, offering synergistic effects when combined with radiotherapy, along with additional benefits in modulating immune responses and reducing inflammation.

Another promising way of radiosensitising cancer cells specifically is by using targeted radionuclide therapy (TRT). Unlike external beam radiotherapy, TRT offers the advantage of specifically targeting affected organs and delivering radiation directly from within the body. TRT employs a vector designed to selectively bind to cancerous cells. This vector is coupled with a radionuclide molecule, which emits radiation through its decay, utilising α particles, β particles, or Auger electrons [215]. While TRT shows significant potential, the integration of nanomaterials introduces challenges, such as increased uptake by the liver and spleen, except when using the intratumoural route. Although studies have successfully achieved high radionuclide coupling efficiencies, the comprehensive review by Reilly et al. highlights a research gap: most studies emphasise imaging properties rather than the radiosensitising effects of such nanocomplexes [216]. However, there are still some examples cited. It appears that 1.85 MBq ^90^Y-IONPs (>97% labelling efficiency) injected intratumourally successfully inhibited tumour growth; it was also observed when combined with hyperthermia in models of colon carcinoma and mammary carcinoma [216]. Future research should then focus on how IONPs coupled with targeted radionuclide could synergise for radiosensitising cancer cells. Such efforts would provide deeper insights into the intersection of ferroptosis, nanotechnology, and TRT, ultimately paving the way for more effective and targeted therapies.

Consequently, despite the promising advances and potential of metal-based nanoparticles and ferroptosis-inducing strategies, further research is required to bridge the gap between in vitro findings and clinical application. The integration of advanced models such as organoids and PDXs is proving pivotal in validating these innovative approaches, with encouraging evidence supporting their role in targeting ferroptosis pathways and enhancing radiosensitivity.

## 7. Concluding Remarks

The integration of nanotechnology has demonstrated significant advancements, notably through the application of metallic nanoparticles, including IONPs. These nanoparticles exhibit several properties, such as superparamagnetism, biocompatibility, and modifiable surfaces, making them well suited for various biomedical applications, including MRI contrast enhancement, drug and gene delivery, and techniques like magnetic hyperthermia and magnetic particle imaging.

One of the main applications of IONPs is drug delivery. Their unique properties enhance drug bioavailability, improve targeting precision, and reduce systemic toxicity, thus providing a substantial therapeutic advantage over traditional treatments like cisplatin alone. The functionalisation of IONPs with specific ligands facilitates targeted delivery and improves their accumulation in tumour tissues, leading to more effective treatment outcomes with minimised adverse events.

Moreover, the induction of ferroptosis via IONPs offers a novel therapeutic strategy for overcoming radio- and chemoresistance. Ferroptosis, an iron-dependent form of regulated cell death, has shown potential in selectively targeting CSCs, which often evades conventional therapies. By inducing ferroptosis, IONPs may render cancer cells more susceptible to radiotherapy, thus enhancing overall treatment efficacy.

The combination of IONPs with radiotherapy has also shown promise as a means of improving therapeutic effects. Research indicates that IONPs can act as radiosensitisers by amplifying radiation effects through increased production of reactive oxygen species (ROS), lipid peroxidation, and modulation of the immune response. This latter capability may shift the tumour microenvironment toward a more pro-immunogenic state, contributing to improved treatment outcomes. Also, their ability to address critical challenges, such as radiotherapy resistance, underscores their potential in developing future therapeutic strategies. However, ongoing research and clinical trials are essential for further validating these approaches and refining their application in personalised medicine.

Despite the potential of ferroptosis, challenges remain in its characterisation, particularly concerning the role of iron in lipid peroxidation and the involvement of organelles like mitochondria. Comprehensive preclinical models are needed to elucidate the biochemical pathways and cellular responses associated with ferroptosis, especially in conjunction with radiotherapy. Key areas of ongoing research include understanding mitochondrial contributions to ferroptosis and identifying reliable biomarkers and optimal conditions for inducing this form of cell death in resistant cancers.

HNCs share many similarities with other types of cancer, yet they also possess distinct features that set them apart. Like all cancers, HNCs are influenced by the 14 specific hallmarks of cancer. The first six, described in 2000 and the most well known, include a lack of responsiveness to suppressive signals and sustained proliferative signalling, ability to evade apoptosis and replicative immortality, as well as angiogenesis, invasion, and metastasis. HNCs, like many other malignancies, arise due to common carcinogens, notably alcohol and tobacco consumption, which also contribute to cancers of the oesophagus, lungs, breast, and liver. Treatment approaches including surgery, chemotherapy, and radiotherapy are similar across HNCs and different other cancer types. Various signalling pathways play a role in cancer progression, and ferroptosis has recently emerged as a potential vulnerability. This unique form of cell death offers a novel therapeutic target that may help overcome treatment resistance.

However, what makes HNCs particularly challenging is their anatomical complexity. The affected regions encompass critical structures of the respiratory and digestive systems, as well as the vocal cords, making surgical interventions highly delicate and often leading to severe comorbidities, such as impaired speech, swallowing, or breathing. Furthermore, HNC patients frequently face high recurrence rates due to the continuous exposure of these areas to carcinogens. These unique challenges underscore the need for specialised treatment strategies that balance oncologic control with preserving function and quality of life.

While IONPs represent a promising avenue for radiosensitisation through ferroptosis induction in several cancer types, additional work remains to optimise their clinical applications, particularly for HNCs. Issues such as nanoparticle retention, circulation time, and biodistribution must be addressed. Nevertheless, nowadays, the synergistic approach of combining IONPs with radiotherapy through the induction of ferroptosis presents an exciting opportunity to enhance treatment outcomes for HNCs, potentially reducing toxicity while effectively targeting resistant cancer cells.

## Figures and Tables

**Table 1:** Survival probabilities associated with targets from the ferroptotic pathway in HNCs. FPN1 (SLC40A1): ferroportin 1 and solute carrier family 40 member 1, responsible for exporting Fe^2+^ PCBP1: poly(rC)-binding protein 1, NCOA4: nuclear receptor coactivator 4, FTH1: ferritin heavy chain 1, TXNRD1/2: thioredoxin reductase 1/2, SLC7A11: solute carrier family 7 member 11, facilitating glutamine transport, FSP1 (AIFM2): ferroptosis suppressor protein 1 (apoptosis-inducing factor mitochondria associated 2).

Molecular Pathway	RNA Levels	Survival Probability
Iron metabolism	FPN1 (SLC40A1)	High expression associated with better survival (*p* < 0.05)
Iron metabolism	PCBP1	High expression associated with poorer survival (*p* < 0.01)
Iron metabolism	NCOA4	High expression associated with better survival (*p* < 0.05)
Iron metabolism	FTH1	High expression associated with poorer survival (*p* < 0.001)
Antioxidant defences	TXNRD1	High expression associated with poorer survival (*p* < 0.05)
Antioxidant defences	TXNRD2	High expression associated with poorer survival (*p* < 0.01)
Glutamine pathway	SLC7A11	High expression associated with poorer survival (*p* < 0.05)
Glutamine pathway	FSP1 (AIFM2)	High expression associated with poorer survival (*p* < 0.05)

**Table 3:** Synoptic overview of the different platforms discussed in this chapter.

IONPs Structure	Molecule Delivered	Cancer Model	Outcome	References
Folic acid (FA)- and intracellular aggregation ability peptide-coated IONPs	Cisplatin	HNE-1 cells and HNE-1 cisplatin-resistant cells	Significant Reduction in half-maximal inhibitory concentration (IC_50_) in both cell lines compared to cisplatin alone; Increase in ROS generation; Increase in apoptosis rate.	[133]
TAT-PEG_2000_-coated IONPs	Cisplatin	HNE-1 cisplatin-resistant cells and CNE-2 cisplatin-resistant cells	Significant Reduction in half-maximal inhibitory concentration (IC_50_) in both cell lines compared to cisplatin alone; Improved cellular uptake compared to non-peptide IONPs.	[157]
Polyacrylic-coated mesoporous IONPs	Bleomycin	Cal-27 and CNE2 cell lines Cal-27 xenograft mice	Significant Induction of apoptotic figures; Reduction in in vivo tumour growth rate.	[158]
Polyethyleneimine-coated IONPs	Tumour necrosis factor-related apoptosis-inducing ligands	Tca83 cell line Tca83 xenograft mice	Significant Induction of apoptotic figures in vitro; Reduction in in vivo tumour growth rate.	[159]

**Table 4:** Effects of metallic nanoparticles combined with radiotherapy. This table presents a non-exhaustive list of cancer therapies using metal-based nanoparticles (NPs) and the effects induced by their combination with radiotherapy (RT).

Type of Metal	Cancer Model	Effects of the Combination of NPs and RT	Reference
Hafnium oxide	Glioblastoma Colorectal cancer Acute monocytic leukaemia Breast cancer	Increase in lysosomal membrane permeabilisation Increase in lipid peroxidation Increase in ferroptosis	[188]
Gadolinium	Triple-negative breast cancer	Inhibition of the pathway NRF2-GSH-GPX4	[190]
		Increase in ferroptosis	
Iron oxide	Lung carcinoma	Decrease in survival fraction Decrease in TrxR activity	[13]
Gold	Lung carcinoma	Decrease in survival fraction	[191,192]
		Decrease in TrxR activity	
Iron oxide nanocluster	Lung cancer	Increase in lipid peroxidation and ROS Increase in ferroptosis and apoptosis Significant decrease in tumour volume	[193]
Hyaluronic acid-based nanoparticles with iron	Lung carcinoma Melanoma	Increase in DNA damage Increase in the level of ROS Increase in cell death	[194]
Fe(III)-polydopamine core and platinum shell, covered with hyaluronic acid	Breast cancer	Decrease in viability Increase in ROS Depletion in GSH in the presence of light Decrease in tumour volume in vivo	[195]
Nanocomplexes (ferrous oxide or copper oxide)	Glioma stem cell	Increase in ferroptosis	[196]

## Data Availability

Data sharing is not applicable.
